# The Neglected Sibling: NLRP2 Inflammasome in the Nervous System

**DOI:** 10.14336/AD.2023.0926-1

**Published:** 2024-05-07

**Authors:** László Ducza, Botond Gaál

**Affiliations:** Department of Anatomy, Histology and Embryology, Faculty of Medicine, University of Debrecen, Hungary, Hungary

**Keywords:** inflammasome, NLRP2, neurological disorders, connectome, STRING

## Abstract

While classical NOD-like receptor pyrin domain containing protein 1 (NLRP1) and NLRP3 inflammasomal proteins have been extensively investigated, the contribution of NLRP2 is still ill-defined in the nervous system. Given the putative significance of NLRP2 in orchestrating neuroinflammation, further inquiry is needed to gain a better understanding of its connectome, hence its specific targeting may hold a promising therapeutic implication. Therefore, bioinformatical approach for extracting information, specifically in the context of neuropathologies, is also undoubtedly preferred. To the best of our knowledge, there is no review study selectively targeting only NLRP2. Increasing, but still fragmentary evidence should encourage researchers to thoroughly investigate this inflammasome in various animal- and human models. Taken together, herein we aimed to review the current literature focusing on the role of NLRP2 inflammasome in the nervous system and more importantly, we provide an algorithm-based protein network of human NLRP2 for elucidating potentially valuable molecular partnerships that can be the beginning of a new discourse and future therapeutic considerations.

## 1. Introduction

Acute inflammation is a physiological defense mechanism in response to pathogens and cell damages; chronic inflammation, however, is considered as a dysregulated maladaptive clinical phenomenon without any recuperative benefits [1]. Chronic inflammation has been associated with many neurological disorders, [2] thus studying inflammasomes is of particular importance.

Classically, the currently known canonical inflammasomes (NLRP1, NLRP2, NLRP3, NLRP6, NLRP7, NLRP9, NLRP12, absent in melanoma 2 (AIM2) and pyrin inflammasomes) recruite caspase-1 enzyme, cleaving the zymogen interleukin-1β (IL-1β), IL-18 or IL-37 to induce lytic pyroptotic cell death and subsequent inflammatory downstream signaling [3-7].

Inflammasome machinery was first characterized in macrophage lineage cells by Martinon et al [8] in 2002, functioning as a caspase-activator normally inactive intracellular protein platform releasing mature proinflammatory mediators upon activation by microbial antigens and foreign and/or host-derived danger factors. Since then inflammasomes have been identied in several cell types, they were found partake in the activation of innate immune system in numerous organs including the central nervous system (CNS) as well. Innate immunity is the major core component of neuroinflammation regarding both causative and consequential viewpoints of neuropathologies [9, 10, 11]. Nowadays, it is undoubtedly accepted that neuroinflammatory- then neurodegenerative states are the aftermaths of abnormal protein aggregations such as amyloid-β, α-synuclein or prions that may directly induce inflammasome- assembling [12].

**Figure 1.**
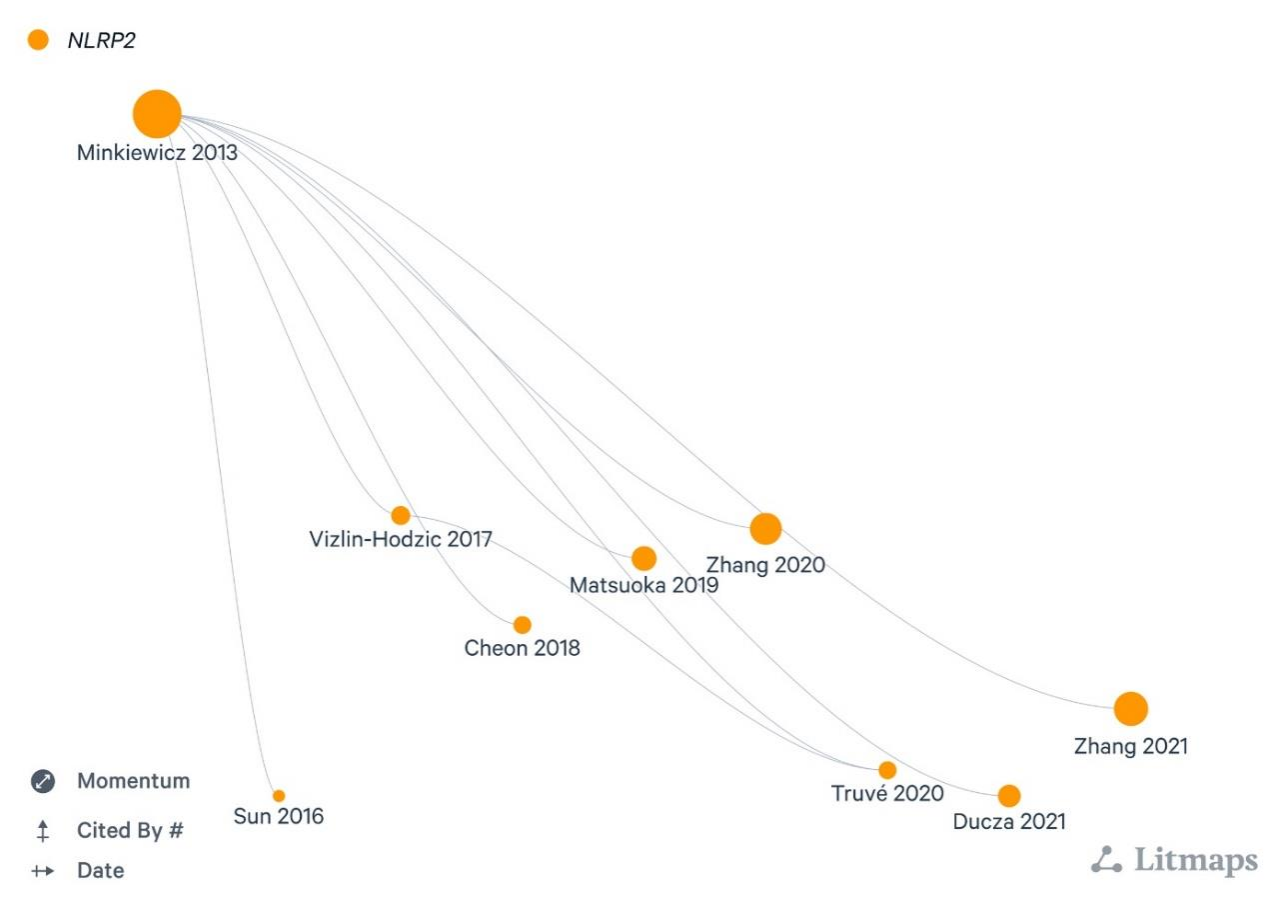
**Litmaps visualisation of original research articles targeting NLRP2 inflammasome in the nervous system Articles are shown in display mode “Momentum”**. Calculation is based on "Cited By" count, weighed by recency.

**Figure 2.**
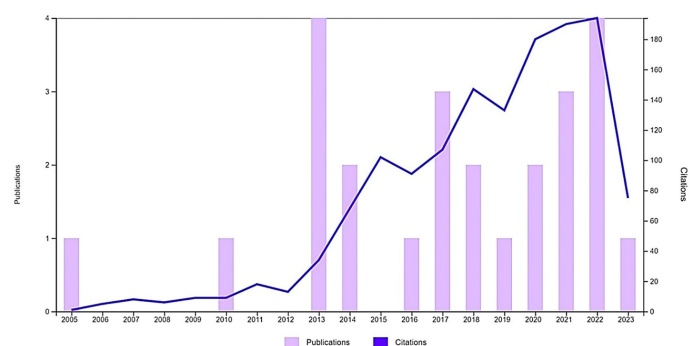
Number of publications and citations related to NLRP2 inflammasome in the nervous system (2005-2023) based on Web of Science Core Collection (WOSCC).

NLRP2 (alternate names: NALP2, PYPAF2, NBS1, PAN1, CLR19.9) inflammasome is an intracellular multimer protein signaling hub assembled by three main elements: (i) a sensory component, termed NLRP, involved in the recognition of Pathogen-Associated Molecular patterns (PAMPs) and Damage-Associated Molecular Patterns (DAMPs), (ii) an adaptor unit, termed Apoptosis-associated Speck-like protein containing a caspase-activation and recruitment domain (ASC, CARD) as well as the (iii) effector caspase-1 enzyme [3, 8-16]. NLRP2 activation recruites „function to find” (FIIND) and a CARD-containing protein Cardinal that interacts with caspase-1 [15]. Of note, NLRP2 was earlier shown to govern inflammasome signaling by inhibiting nuclear factor kappa B (NF-κB) transcription factor [17, 18]. Recent data have indicated that NLRP2 robustly increases the amount of NF-κB regulated cytokines in cystinosis [19]. Moreover, several lines of evidence support the critical regulator role of NLRP2 in the reproductive system, hence *NLRP2* gene was reported as one of the mammalian maternal effect genes associated with murine embryogenesis, age-related maternal fertility and idiopathic recurrent miscarriage, respectively [20-22]. Besides, NLRP2 expression was also proven to associate with arsenic-induced skin lesion, chromosomal damage, and respiratory disorders as well [23]. Intriguingly, despite the aforementioned past research on non-neural tissues, our gap of knowledge regarding NLRP2 and its connectome in neuropathological context has still yet to be improved (Fig. 1, Fig. 2).

Although our comprehension of the role of certain inflammasomes in CNS disorders has substantially improved throughout the past two decades, the causes of multiple number of NLRP2- related neurological disorders are indispensable to be further researched. Therefore, first, our study focused on the past experiments related to NLRP2 inflammasome in human and rodent models by providing a state-of- the-art of the literature. Secondly, a publicly available cutting-edge biomedical database called Search Tool for the Retrieval of Interacting Genes/Proteins (STRING, ver.11.5; string-db.org) [24] was employed to envisage potential protein interactions of NLRP2.

Apart from a few exceptions, currently there are future directions neither in NLRP2 research pipelines nor in therapeutic avenues. In this overview, following clarification of the main aspects of NLRP2 research, we aimed to describe a set of proteins (connectome) that had been already identified in a broad spectrum of brain disorders, but still none of their actions were directly linked with NLRP2. Eventually, we found 15 proteins that may associate with NLRP2, representing promising therapeutic targets and signaling pathways in NLRP2 associated neurological disorders.

## 2. Cellular distribution of major inflammasomes in the nervous system

CNS parenchyma has a remarkably complex architecture represented by diverse and highly interconnected cell types comprising neurons and glial cells (mainly astrocytes, microglia, oligodendrocytes) that collaborate to provide ideal circumstances for proper neuronal development and function. Adding another depth of complexity, in the vast majority of brain disorders not exclusively neurons, but glial- and even peripheral immune cells (granulocytes, monocytes, and lymphocytes) are recruited as well, all endowed with great level of heterogeneity including dynamically changing phenotypes upon noxious stimuli. These cells can vary spatiotemporally in their morphological and ultrastructural features as well as their genetic and protein signature. Complex neuron-glia-immune cell interactions even aggravate the determination of causative relationships and identification of primary disease vs. secondary “reactive” states [25, 26, 27]. Expression of inflammasomal NLRPs have been meticulously investigated in the nervous system. Main expression patterns are as follows: NLRP1 and NLRP3: primarily in microglial cells [28, 29], NLRP2 and NLRP3: in astrocytes [7, 30-35], NLRP2: dorsal root ganglia (DRG) cells and neural stem cells [36, 37], AIM2 and NLRP1: in neurons [38, 39]. Recently, oligodendrocytes were also shown to induce NLRP3 activation upon glycolytic stress in Alzheimer’s disease (AD) [40]. Of these types, the distribution of NLRP1 and NLRP3 inflammasomes is the most abundant in the CNS, they play a critical role in neuroinflammation as well as wide range of neurological abnormalities like ischaemic stroke, traumatic brain- and spical cord injury, neurodegenerative diseases, epilepsy or variety of brain infections [11, 41-44].

## 3. NLRP2 expression in the nervous system

### 3.1 Human NLRP2

For many years it remained elusive whether NLRP2 could be validated as a functional inflammasome in the human nervous system. The pioneering milestone was achieved by Julia Minkiewicz et al. in 2013 [7] by verifying the significance of NLRP2 inflammasome in human cortical astrocyte cultures. They proposed that NLRP2 interacted with ionotropic purinergic receptor X7 (P2X7) receptor and pannexin 1 (PNX1), hence mimicking inflammation with ATP stimulation rapidly resulted in caspase-1 activation and subsequent IL-1β release. Conversely, pharmacological blockade of astrocytic PNX1 and P2X7 receptor with probenecid and Brilliant Blue G (BBG) diminished the ATP-induced NLRP2 activation. In addition, siRNA-based silencing of *NLRP2* was also found to disrupt caspase-1 activation, emphasizing its pivotal role in astrocyte immune responses. *NLRP2* gene dysregulation was shown to be involved in human fetal brain development indicating a robust difference between cases of bipolar disorder and healthy individuals [45, 46]. In line with this, Truvé et al. [37] in 2020 found considerable increase of NLRP2 expression in neural stem- and mature cells of patients suffering from bipolar disorder.

### 3.2 Rodent NLRP2

To date, still more NLRP2 related experimental data are accessible from rodent models. Several rodent models are continously used in drug development pipelines, but regrettably the limitations of these concepts are major hindrances to translation attempts from bench to bedside. Few years previously Sun et al. in 2016 [32] have already determined NLRP2 distribution at specific areas of the murine CNS. Low constitutive NLRP2 levels were observed in the cortex, hippocampus and striatum, which were significantly elevated following occlusion of middle cerebral artery mimicking ischemic stroke. Similar results were obtained in primary astrocyte cultures upon oxygen-glucose deprivation. These findings were further corroborated by Cheon et al. in 2018 [33], who searched the interaction of apoptosis signal-regulating kinase 1 (ASK1) protein with astroglial NLRP2 inflammasome after ischemic stroke. ASK1 inhibition effectively downregulated NLRP2, as well as proinflammatory cytokine release in murine model and cultured astrocytes. In former studies, NLRP1 and NLRP3 inflammasomes were recognised in neuropathic models and spinal injury [47-50], but only scanty data were provided about NLRP2 protein following peripheral inflammation. Matsuoka and his colleagues in their study [36] characterised NLRP2 inflammasome in NeuN- and peripherin positive DRG cells of C-fibers implying its significant role in nociceptive processing. They found that both siRNA gene silencing of NLRP2 expression and pharmacological blockade of caspase-1 prevented nociceptive hypersensitivity, measured in two peripheral inflammatory models using either complete Freund adjuvant (CFA)- or ceramide. Their data did not support possible activation of matrix metalloproteinase enzyme 9 or other inflammasome types except NLRP2, therefore they hypothesized that caspase-1 enzyme in DRG neurons worked presumably in NLRP2-dependent manner. Enigmatically, inhibition of caspase-1 and siRNA silencing of NLRP2 attenuated mechanical, but not thermal hypersensitivity upon inflammation. This finding can be explained by the low NLRP2 level in thermal sensor transient receptor potential vanilloid subtype-1 positive neurons.

Our workgroup also investigated the putative changes of NLRP2 expression in CFA- induced inflammatory pain model within the spinal dorsal horn [31]. Although, NLRP1, NLRP2 and NLRP3 inflammasomes have been all detected in CNS [46, 51], our results showed that dorsal horn astrocytes mainly expressed NLRP2, colocalisation was entirely lacking between NLRP1 and astrocytes, we did not investigate DRG cells. With protein blot analysis we also verified a robust increase of NLRP2 protein in spinal cord tissue lysates upon CFA injection. Not unexpectedly, the role of astroglial NLRP2 has also been reported in chronic mild stress induced depression [34], where they introduced an intraperitoneal injection of tryptophane derived metabolite kynurenine (Kyn), which upregulated NLRP2 in hippocampal primary astrocytes cultures. Simultaneously, Kyn treatment aided nuclear translocation of NFκB to promote *NLRP2* transcription, eventually leading to inflammasome activation. These findings also illustrated that Kyn elicited depressive behaviour was eliminated following astrocytic *NLRP2* knockdown, proposing a crucial role for this inflammasome in depressive behaviours.

Recently, the role of NLRP2 inflammasome has been elucidated in murine AD [35]. In this study, glucagon-like peptide-1 (GLP-1) receptor agonist exenatide attenuated neuroinflammation in piriform cortex, resulting in cognitive improvement of 5x FAD transgenic mice. Additionally, upon exendine-4 treatment of cultured astrocytes, lower amyloid β_1-42_ level was detected confirming the reduced inflammation and oxidative stress presumably via downregulation of NLRP2 inflammasome.

### 3.3 Controversial findings of NLRP2 expression

Taken together, in agreement of results obtained from human and rodent models, expression of NLRP2 inflammasome is relatively scanty, therefore it is less studied in CNS compared with NLRP1 and NLRP3. Furthermore, distribution of NLRP2 appears to be more scattered, hence it has also been detected in different types of astrocytes (such as primary astrocyte cultures or astrocytes of spinal dorsal horn tissue), and also in neurons (such as DRG cells or neural stem cells).

However, the question arises, what the reason could be for the simultaneous duplicated expression in dorsal horn astrocytes and DRG cells during CFA induced inflammatory pain Unfortunately, although there is limited information from experimental approaches, we hypothesize that NLRP2 function may span multiple cell types and induce/influence signalings that require further investigation including data mining techniques.

## 4. Human NLRP2 connectome with STRING database

The complex nature of cells derives from a wide variety of protein-protein interactions. Knowledge of major interactions shows an increasing trend, but novel interactions should also be explored. Meanwhile the information is frequently divided across databases with differing profiles, STRING [24] is a freely accessible online biological web resource that aims to collect protein-protein associations taken from many organisms in a systematic, yet quality-controlled way. STRING greatly facilitates access to a plethora of physical and functional interactions from several sources: literature text mining, computated predictions related to co-expression with genomic context as well as high-throughput experimental data from curated resources. Subsequently, all of these interactions undergo careful evaluation and scoring. STRING networks comprise precomputed protein clusters hierarchically organising the full STRING network employing an average linkage algorithm.

**Figure 3.**
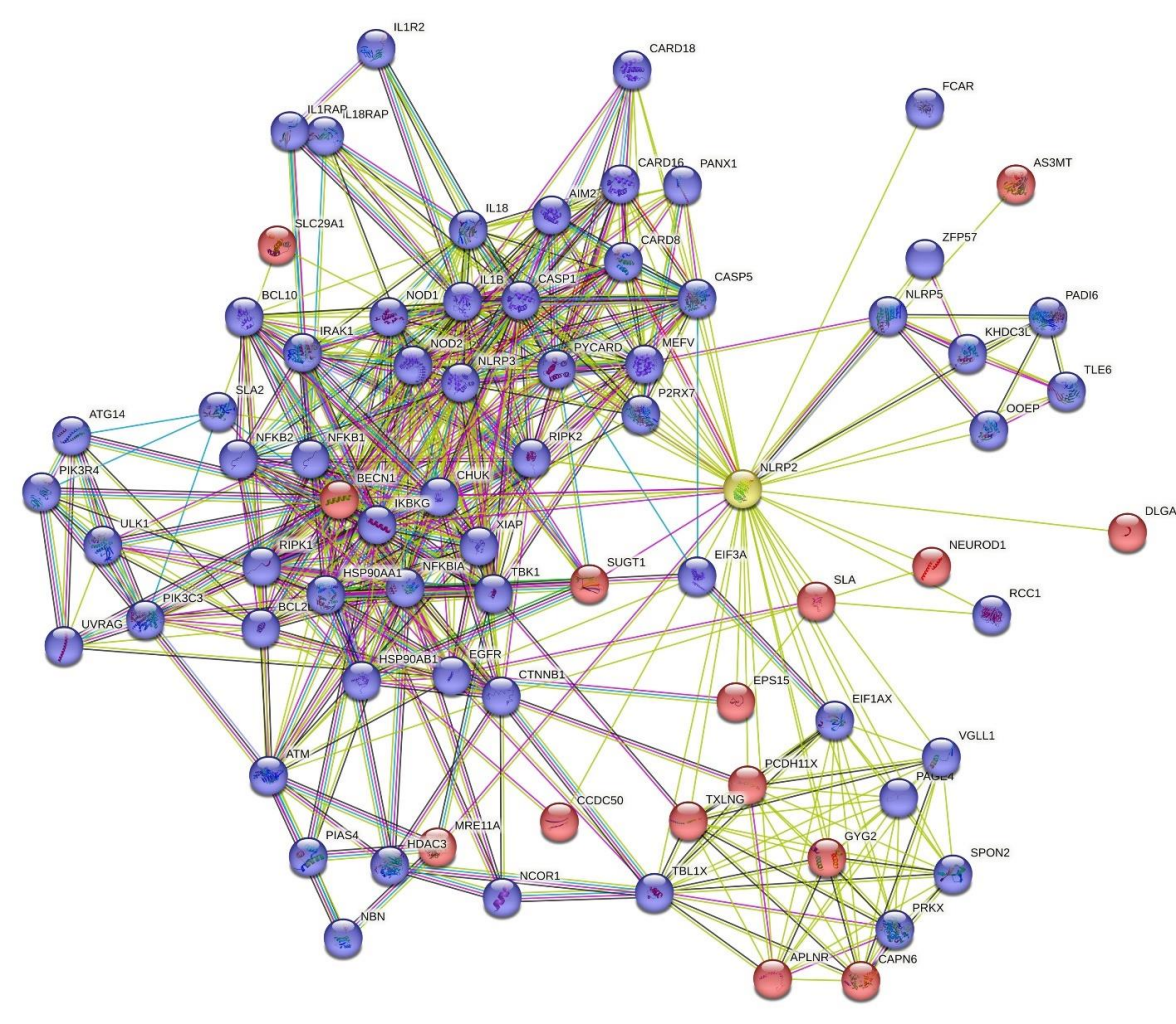
**The extended STRING connectome of NLRP2 protein**. Color code is as follows: known interactions: light blue, from curated databases; magenta, experimentally determined; predicted interactions: green, gene neighbourhood; dark blue, gene co-occurrence; others: lime, text-mining; black, coexpression; purple, protein homology. Red colour indicates the selected 15 nodes potentially involved in neuropathologies.

In our case the query service of the platform (ver.11.5, string-db.org) was initiated with the following input: “*NLRP2*”, species: “*Homo sapiens”.* NLRP2 connectome was generated by our arbitrary parameters that were as follows: full-STRING network (containing both functional and physical interactions), evidence-based network edges (types of interactions are shown with different line colors) with a cut-off interaction confidence score: 0.4. For data visualisation all the interaction sources were collected: textmining, experiments, databases, coexpression, neighbourhood, gene fusion, co-occurence.

Based on our settings the network statistics of NLRP2 connectome involve 76 nodes (with an average node degree: 13.3), 504 edges, with a PPI enrichment p-value < 1.0e-16 (Fig. 3). From the direct interactions of NLRP2 (45 nodes, Table 1), possible functional partners (15 nodes) identified by literature overview using WOSCC (of the 45 proteins only those hits were selected, which were earlier investigated with relevant role in brain disorders at least in one article), are introduced in the order of their interaction score (chapter 4.14-4.15). We excluded those items that were either experimentally investigated with NLRP2 protein in the nervous system or reviewed in chapter 1-3.

The detailed principles of how scoring is precisely calculated are legible on the string.db website. Briefly, STRING determines the numerical value of the interaction score by combining the probability values from all of the evidence channels and makes correction for the probability of randomly occurring interactions applying a freely available python script (combine_ subscores. py).

**Table 1:** List of direct interactions of NLRP2.

	Node protein hits	Full name	Interaction score
**1**	CASP1	Caspase-1	0.862
**2**	PYCARD	Apoptosis-associated speck-like protein containing a CARD	0.825
**3**	CASP5	Caspase-5	0.811
**4**	MEFV	Pyrin	0.798
**5**	CARD8	Caspase recruitment domain-containing protein 8	0.771
**6**	SLA	Src-like-adapter; Adapter protein	0.752
**7**	KHDC3L	KHDC3-like protein; KH domain containing 3 like, subcortical maternal complex member	0.739
**8**	BECN1	Beclin-1	0.664
**9**	AIM2	Interferon-inducible protein AIM2	0.645
**10**	P2RX7	P2X purinoceptor 7; Receptor	0.638
**11**	CARD16	Caspase recruitment domain-containing protein 16	0.629
**12**	PANX1	Pannexin-1	0.600
**13**	IL18	Interleukin-18	0.597
**14**	SLA2	Src-like-adapter 2; Adapter protein	0.590
**15**	PAGE4	P antigen family member 4	0.575
**16**	EIF1AX	Eukaryotic translation initiation factor 1A	0.567
**17**	IL1B	Interleukin-1 beta	0.563
**18**	ZFP57	Krab domain-containing zinc finger protein	0.563
**19**	VGLL1	Transcription cofactor vestigial-like protein 1	0.562
**20**	CHUK	Inhibitor of nuclear factor kappa-B kinase subunit alpha	0.560
**21**	NLRP5	NACHT, LRR and PYD domains-containing protein 5	0.556
**22**	PCDH11X	Protocadherin-11 X-linked; Potential calcium-dependent cell-adhesion protein	0.547
**23**	GYG2	Glycogenin-2	0.515
**24**	OOEP	Oocyte-expressed protein homolog	0.514
**25**	MRE11A	Double-strand break repair protein MRE11	0.490
**26**	TLE6	Transducin-like enhancer protein 6	0.487
**27**	SUGT1	SGT1 homolog, MIS12 kinetochore complex assembly cochaperone	0.485
**28**	RIPK2	Receptor-interacting serine/threonine-protein kinase 2	0.473
**29**	SPON2	Spondin-2	0.471
**30**	IKBKG	NF-kappa-B essential modulator	0.470
**31**	EPS15	Epidermal growth factor receptor substrate 15	0.468
**32**	NEUROD1	Neurogenic differentiation factor 1	0.466
**33**	CAPN6	Calpain-6	0.462
**34**	PRKX	cAMP-dependent protein kinase catalytic subunit PRKX	0.456
**35**	TXLNG	Gamma-taxilin	0.455
**36**	DLGAP2	Disks large-associated protein 2	0.449
**37**	APLNR	Apelin receptor	0.445
**38**	SLC29A1	Solute carrier family 2	0.444
**39**	CARD18	Caspase recruitment domain-containing protein 18	0.437
**40**	AS3MT	Arsenite methyltransferase	0.423
**41**	CCDC50	Coiled-coil domain-containing protein 50	0.421
**42**	TBL1X	F-box-like/WD repeat-containing protein TBL1X	0.418
**43**	PADI6	Protein-arginine deiminase type-6	0.418
**44**	FCAR	Immunoglobulin alpha Fc receptor	0.408
**45**	RCC1	Regulator of chromosome condensation	0.400

### 4.1 Src-like-adapter proteins (SLA proteins)

SLA proteins are signal transducing proteins received their name after the protooncogen Src family tyrosine kinases (STK). Unlike Src kinases, SLA proteins do not have tyrosine kinase activity, yet they contribute to multiple cellular processes. Specifically, the SLA protein 1 (SLA1) was recognised earlier in proteosomic degradation of redundant N-methyl-D-aspartate (NMDA) receptors preventing excitotoxicity [52]. Furthermore, STKs were also investigated because of their role in central sensitisation, hence intrathecal injection of STK inhibitors mitigated mechanical allodynia in chronic pain models [53]. In addition, STK inhibitor PP2 could efficiently downregulate lipopolysaccharide (LPS)- induced microglial activation and production of neuroinflammatory factors via NF-κB inhibition in MPTP-induced murine model of Parkinson’s disease (PD) [54, 55].

### 4.2 Beclin 1 (BECN1)

BECN1 is a multifaceted protein, having a well-established role in an apoptotis-related multimer complex, which is essential for nucleation of autophagic vesicles [56-58]. Convincing data earlier confirmed the contribution of BECN1 to neuroinflammation [59], associated with extracellular increase of Aβ plaques as well as microglia-derived proinflammatory cytokines. Similarly, disruption of autophagy via decrease of BECN1 ultimately leads to exaggerated microglial release of IL-1β and IL-18. In patients suffering from Alzheimer’s disease (AD) microglial cells exhibited strongly reduced level of BECN1 [60, 61]. In addition, beneficial effect of BECN1 overexpression was unveiled in murine huntingtin (HTT) model of Huntington’s disease (HD). They concluded that in early disease stage BECN1 administration restored network impairments occurred accumulation of mutant HTT aggregates [62].

### 4.3 Protocadherin-11X (PCDH11X)

Protocadherins (PCDHs), as the most versatile cadherin proteins, are involved in several neural events such as synaptogenesis, dendrite- and axon growth. They are classified into three main α, β, and γ gene clusters with approximately 60 genes extended with 10 non-clustered δ genes [63]. It is a well-known fact that *PCDH* gene dysregulations may result in neurological disorders, especially HD and AD. In knock-in murine model of HD, RNA-seq experiments performed by Langfelder et al. [64] determined 4 gene modules associated with 37 clustered *PCDH* genes together with non-clustered δ members *PCDH20* and *PCD11X/Y*. Despite the fact that Carrasquillo and his group [65] earlier reported 4 single nucleotide polymorphisms (SNPs) of *PCD11X* (SNPs: rs5984894, rs2573905, rs5941047, rs4568761) with hypothesized role in late-stage AD, later works with thorough genome analysis revealed no correlation between these SNPs and AD [66, 67].

### 4.4 Glycogenin-2 (GYG-2)

Glycogen is a primarily used glucose reservoir that provides rapidly accessible energy, participating in blood sugar control as well as muscle contraction. Glycogenesis requires the cooperation of both GYG glycosyltransferase and glycogen synthase (GS) enzymes. GS is allosterically activated by glucose-6-phosphate, but inhibited by glycogen synthase kinase (GSK) phosphorylation [68, 69]. Defects of glycogen metabolism, specifically the function of GYG, may lead to a genetically heterogenous and rapidly progrediating neurodegenerative disorder called Leigh syndrome that displays severe bilateral pathological alterations in cerebellum, spinal cord and brainstem. *In vitro* experiments earlier unveiled that human hemizygous missense mutation of *GYG-2* gene impaired self-glycosylation leading to disruption of glycogenesis [70]. Another glycogenesis related abnormality, the Lafora disease is characterised by aberrant intracellular glycogen deposits in both neurons and astrocytes [71]. Recently, ubiquitous serine/threonine GSK-3 enzyme has become an important player in neuronal metabolism. GSK-3 acts as the main negative regulator of GS, its pathological role has been already confirmed in AD. GSK-3 targets include Aß and tau hallmark proteins of senile plaques and neurofibrillary tangles [72], confirmed by the significant increase of GSK-3 level in postmortem brain of AD patients [73]. Moreover, recent findings have demonstrated that lysophosphatidic acid, an identified GSK-3 activator, showed association with AD biomarkers Aß, total tau and phospho-tau [74].

### 4.5 Meiotic recombination 11A (MRE 11A)

Undoubtedly, genome maintenance is vital in every cell, by preventing potential DNA damage via sophisticated DNA repair mechanisms [75]. Considering that neurons are post-mitotic cells producing high number of free radicals through their active metabolism, unlike dividing cells, they can not use the machinery of homologous recombination to repair double-strand break (DSB) in an error-free manner. Thus, they rather recruit error-prone repair methods like nonhomologous end joining [76]. Of the several types of DNA damages, DSBs are regarded the most deleterious for neurons, worsening their function and survival. Owing to the fact that no complementary strand template exists for repair, genome instability, and ultimately age-related cognitive disorders and neurodegeneration may develop [77, 78]. Given its key determinant role in MRE11-RAD50-NBS1 (MRN) damage sensor complex, MRE11 orchestrates ATM- or ATR-mediated DNA damage responses upon DSBs [79]. MRE11 nuclease defects were found to be associated with human pathologies such as progressive myoclonic ataxia [80], Nijmegen breakage syndrome-like disorder [81] and Ataxia-Telangiectasia-like disorder [82]. Furthermore, role of MRE11 was also revealed in microcephaly, cognitive disabilities and cerebellar degeneration [83], but so far only one article reported subtantially reduced level of MRE11 and other members of MRN complex in AD patients [84].

### 4.6 Suppressor of G2 allele of Skp1 (SUGT1)

SUGT1 is a major component of the CBF3 kinetochore as well as SCF ubiquitin ligase complexes [85, 86]. SUGT1 binds heat shock protein 90 (Hsp90) via CHORD-Sgt1 domain, therefore it is also considered as a member of chaperone complexes [87, 88]. Additionally, SUGT1 was found to be associated with innate immunity through Nod1 stabilization [89]. SUGT1 is abundantly expressed in hippocampal and cortical neurons as well as in Purkinje cells and cerebellar glial cells. Unsurprisingly, the significance of SUGT1 was earlier described in neurodegenerative diseases including AD and PD respectively [90, 91]. In AD brain, fewer SUGT1 immunopositive neurons were found compared with control [92], but in contrast, *SUGT1* mRNA level was robustly enhanced in fronto-temporal cortex of human PD [91].

### 4.7 Epidermal growth factor receptor substrate 15 (EPS15)

EPS15 tyrosine kinase substrate was first identified thirty years ago as the binding partner of AP-2 complex member alpha-adaptin, playing a crucial role in clathrin-mediated endocytosis as well as vesicular transport [93, 94]. Given its localisation in coated pits, it was proven that EPS15 contributed to internalization of the epidermal growth factor receptor (EGFR). For instance, antibody microinjection against EPS15 or using *EPS15* knockdown animals inhibited clathrin-mediated endocytosis of EGFR [95-97]. EPS15 participates in the EGF-AKT pathway mediated pro-survival signaling that is highly reduced in PD patients. In accordance with this, striatal dopaminergic neurodegeneration can be prevented upon EGFR activation in PD animal model [98]. Nevertheless, even if EGF-EGFR binding promotes AKT pathway, it also stimulates ubiquitine-mediated receptor endocytosis and degradation. In doing so, ubiquitinated EGFRs, guided by EPS15, are trafficked directly to proteosomal degradation. However, EPS15-EGFR interaction can be inhibited by E3 ubiquitin–protein ligase called Parkin that aids ubiquitination of EPS15 while preventing receptor internalisation [99, 100].

### 4.8 Neurogenic differentiation factor 1 (NEUROD1)

It has been well documented that basic Helix-loop-Helix (bHLH) transcription superfamily, especially proneural bHLH subgroup, termed NEUROD family, is a critical determinant of neuronal differentiation in spinal cord, brainstem, and cerebral cortex [101, 102]. The family consists of four members: NEUROD1, NEUROD2, NEUROD4, NEUROD6 that show overlapping, but not identical expression pattern in the cerebral cortex. In addition, they are substantially expressed not only in prenatal, but in adult cortex, cerebellum, and hippocampus as well [103, 104, 105].

In pathological context, increased level of hippocampal *NEUROD1* has been detected in murine model of environmental stress normalised by antidepressant agomelatine [106]. Rubio-Cabezas et al. [107] identified the first human mutations of *NEUROD1* gene in the form of two homozygous frameshift mutations found in patients with neonatal diabetes and neurological disorders including cerebellar hypoplasia, visual impairment, and sensorineural deafness. *NEUROD1* gene ablation produced murine epilepsy due to excessive neurodegeneration of hippocampal dentate granule cells [108], moreover its reduced level was also detected in R6/2 murine model of HD [109]. Despite its significance, *NEUROD1* expression has been debated in AD patients based on RNA-seq results [110] from APPxPS1 murine model. However, the overexpression of NEUROD1 greatly raised dendritic spine density, improving the spatial memory in hippocampus [111]. Nowadays, novel therapeutic approaches attempt to either block ubiquitin-dependent proteosomal degradation of NEUROD1 to prevent neuronal loss or apply induced pluripotent stem cells- derived hippocampal spheroids [112, 113]. Besides, manipulation of *NEUROD1* gene in AD and ischemic stroke efficiently produced cortical neurons from *in vivo* reprogrammed reactive glial cells [114, 115].

### 4.9 Calpain-6 (CAPN6, CANPX, calpamodulin)

CAPN is a Ca^2+^ regulated intracellular non-lysosomal cysteine protease that cleaves numerous substrates resulting in either degradation or modulation of cell responses. To this date, 16 *CAPN* genes have been identified including *CAPN6* gene that belongs to the non-classic (atypical) *CAPN* genes [116, 117]. Despite the high level of *CAPN6* mRNA during embryogenesis, especially in the first brancial arch, skeletal muscle, bronchial epithelium, kidney- and lung sacs, its expression was robustly attenuated after birth [118-121]. In the last decade CAPN6 has attracted the neuroscientists’ attention, as its overexpression was detected in prion disease-affected sheep brain tissues as well as ischemia induced white matter injury [122, 123]. CAPN6 is yet to be investigated in diseases like AD and PD, nevertheless calpains have already been linked to neurodegenerative hallmark proteins such as Aβ plaque and tau hyperphosphorylation in AD. Notably, calpain contributes to reactive astrocyte transformation, since its pharmacological blockade abrogates the neuroinflammatory activity of both astrocyteand microglial cells meanwhile improving cognitive decline in the 3xTg AD model [124, 125].

### 4.10 γ-taxilin (TXLNG, γ-TXLN)

Members of the taxilin family were first recognised as interactive partners of syntaxins involved in intracellular vesicular transport. The family is composed of α-, β-, and γ-TXLNs sharing a coiled-coil domain at their C-terminal. Meanwhile α- and γ-TXLNs are ubiquitously expressed, β-TXLN is less abundant in skeletal- and heart muscle tissues [126-129]. During cell interphase γ-TXLN has a centrosomal localisation with reduced expression at G2/M transition owing to proteosomal degradation. Recently, γ-TXLN has been proposed to control Never in mitosis A-related kinase A2 mediated centrosome disjunction [130].

It has already been shown that inhibition of ER stress sensor γ-TXLN via GSK-3 signaling activates unfolded protein production leading to apoptosis. Moreover γ-TXLN ablation may induce tau hyperphosphorylation in AD patients [131]. In the past few years, even if large body of evidence [132, 133] supported physiological relevance of the taxilin family, further research on the role of family member γ-TXLN in neuropathology would be of great value.

### 4.11 Disks large-associated protein 2 (DLGAP2)

As a specialised matrix, postsynaptic density (PSD) plays a role in mediating excitatory neurotransmission at the postsynaptic terminal. Discs large scaffold proteins (DLGAPs) of PSD are required for proper function of both ionotropic and metabotropic glutamate receptors contributing to synaptic scaling [134-137]. Regardless of the fact that DLGAP1-4 is ubiquitously expressed in CNS, higher protein levels were found within the brain and DLGAP2 showed abundant expression in cortex, hippocampus, olfactory bulb and striatum, but not in cerebellum or thalamus [138-141]. Evidence is accumulating for its involvement in autism, hence *DLGAP2* knockout mice exhibited aggressivity, social dominance and abberant synaptic dysfunction in orbitofrontal cortex [142]. Epigenetic studies also implied that *DLGAP2* contributed to other neuropsychiatric disorders like schizophrenia and post-traumatic stress syndrome [143, 144]. Unsurprisingly, cross-species analyses verified *DLGAP2* as a major regulator in cognitive decline of AD patients, its low cortical expression was associated with neurodegenerative hallmarks [145].

### 4.12 Apelin receptor (APLNR)

APLNR is a G protein-coupled receptor, highly resembling the structure of angiotensin II receptor, providing a potentially therapeutic target in cardiovascular diseases. Its bioactive ligand apelin was reported to play a crucial role in myriad of physiological actions such as cardiac contractility, neoangiogenesis as well as immunoregulation, nitric oxide-induced vasorelaxation, fluid- and bone homeostasis, human immunodeficiency virus-1 infection, neuroendocrine stress, glucose metabolism, appetite, and drink behavior, respectively [146-155].

Compelling evidence emphasizes the involvement of apelin in neuropathologies as well. For instance, apelin-13 was identified to exert neuroprotective effects on cortical neurons via inhibiting serum deprivation-induced oxidative stress and apoptosis [156]. Apelin also downregulates NMDA receptor-mediated excitotoxicity in rat hippocampus [157]. Recently, apelin-13 has been shown to disrupt Aβ- induced memory deficit in Aβ_25-35_-treated animals [158]. Based on earlier observations of Agostinho, Cunha, and Oliveira [159], exacerbated neuroinflammation, characterised by glial-derived proinflammatory cytokines, facilitates neurodegenerative progression in AD patients’ brain. In this context, apelin-13 certained to be neuroprotective by alleviating ischemic stroke as well as the effects of proinflammatory cytokines [160]. Furthermore, insulin-like growth factor 1 (IGF-1), which exerts inhibitory effect on α-Syn aggregation in rodent PD, was found to be upregulated by apelin [161]. It has recently been reported in SH-SY5Y cell model of PD that apelin-13 attenuates selective 6-OHDA induced impairment of dopaminergic neurons [162, 163]. Additionally, apelin also ameliorates NLRP3 inflammasome resulting in dampened systemic inflammation [164]. Apelin has also been targeted in HD, since PolyQ-HTT, a pathological form of HTT that perturbates postsynaptic plasticity and cytoskeleton organisation, [165] is inhibited by apelin via PI3K/AKT and MAPK/ERK signaling [166]. Aforementioned research by Yang [163] has already demonstrated that apelin-IGF interaction may have significance in PD, corroborated by recent findings identifying IGF in clearance of HTT aggregates in HD [167].

### 4.13 Solute carrier family 29A1 (SLC29A1, ENT1)

SLC is a membrane transporter superfamily with more than 400 members and 65 families mediating translocation of versatile substances across biological membranes, such as electrolytes, lipids, sugars, amino acids, neurotransmitters or drugs [168, 169]. It is worth mentioning that SLCs have been considered significant pharmacological targets, thus developing new agents related to SLCs may aid their bioavailability in drug interactions- and disposition as well as toxicity [170-172]. SLC29 contains four human intrinsic energy independent membrane proteins termed Equilibrative Nucleoside Transporters (hENT1-4). ENTs regulate facilitative bidirectional nucleoside transport showing capability for transporting both pyrimidines and purines [173, 174].

Significance of SLC29A1 (ENT1) adenosine transporter has been highlighted in 2017 when Yu-Han Kao et al. [175] shed light on dysregulation of adenosine metabolism in HD. Striatal ENT1 expression was robustly enhanced in R6/2 and Hhd^150Q^ models and reduced following either intrastriatal injection of ENT1 inhibitor JMF1907 or genetic suppression leading to higher survival rate of R6/2 mice. Later, others [176] found that application of adenosine analogue ENT1 inhibitor J4 was promising in preventing spatial memory decline in murine APP/PS1 model of AD. Moreover, chronic administration of J4 reversed the impaired basic synaptic signaling, long-term potentiation, excitatory synaptic expression and signaling of neuronal plasticity such as GSK-3ß or PKA. Recently, J4 has been tested in murine model of tauopathy, and was found to block tau hyperphosphorylation while improving memory, mitochondrial dysfunction, and synaptic loss [177]. Collectively, the evidence opens new prospects for neuropathological studies with adenosyne dysregulation in mind.

### 4.14 Arsenite methyltransferase (AS3MT)

Inorganic arsenic (iAs) has been found in highest amount in the air as arsenic trioxide (As_2_O_3_), but it may be present in food, water or soil, predominantly in the form of either AsO_3_ or AsO_2_ (arsenite) [178]. Regrettably, an increasing number of people suffer from the consequences of iAs exposure to drinking water. It is particularly a concern in Bangladesh and Eastern parts of India, where arsenic inflicted crisis is a natural calamity affecting approximately 43 million people [179]. Apart from this, the most frequent causes of poisoning include occupational contamination, moonshine alcohol or malevolent delivery [180].

The role of as has robustly emerged in numerous deleterious neurological symptoms such as memory deficit, Guillain-Barre-like neuropathy, encephalopathy, peripheral neuropathy, PD and AD. However, to date, particularly in terms of mechanisms underlying arsenic neurotoxicity, no remarkable success has been achieved. A growing body of experimental evidence proposes that free radicals provoke neroinflammation, mitochondrial dysregulation, ER stress, apoptosis and protein homeostasis that may be implicated in AD [181-183]. Oxidative stress, mostly incriminated in arsenic-induced neuronal damage, substantially elevates the level of reactive oxygen species while reducing expression of superoxide dismutase and glutathione (GSH) [184, 185]. AS3MT contributes to iAs processing in the presence of GSH, thus GSH deficit may prevent detoxification resulting in AD [186]. It is nowadays considered trivial that neuroinflammation is involved in AD pathology [187]. As also appears to activate microglia cells that secrete proinflammatory cytokines [188, 189]. Besides, several data showed toxic effects of as on brain mitochondria [190, 191]. As has also been found to induce apoptosis of cerebellar neurons via activation of JNK and p38MAPK signaling [192]. Recent evidence shows that as upregulates apoptotic Bax and decreases antiapoptotic Bcl-2 factor [193]. Arsenic-mediated posttranslational modifications and impairment of ubiquitination may also damage proteostasis via accumulation of abnormal hallmark protein aggregates [194, 195].

### 4.15 Coiled-coil domain containing protein 50 (CCDC50)

Autophagy is a phylogenetically conserved lysosomal degradation program that coordinates lysosomal fusion with autophagosomes [196]. Mutations or deficiencies associated with autophagy highlight their significance in exacerbated inflammatory disorders [197]. CCDC50, also termed as Ymer, functions as an ubiquitous autophagy receptor that supresses inflammatory responses by disrupting NLRP3 inflammasome [198, 199]. Previous findings have reported that CCDC50 also prevents ligand-induced downregulation of EGFR as well as regulate NF-κB, Fas and interferon signaling. Thus, dysregulation of CCDC50 may play a pivotal role in clinical implications such as viral infection and autoinflammatory disorders including systematic lupus erythematosus [200].

Albeit insufficient data are yet available on CCDC50 in the context of neurodegeneration, genome wide association studies showed that SNPs of 3q28 locus were correlated with tau/ptau levels of cerebrospinal fluid and a broad range of AD fenotypes. However, no significant SNPs were found in *CCDC50* and other genes such as *GEMC1, OSTN, SNAR1, IL6RAP or UTS2D* from the same locus, they are substantially expressed in brain tissue and contribute to synaptogenesis [201].

## 5. Discussion and future prospects

To date, neurodegenerative disorders lack effective therapeutic agents or programs to prevent, efficiently influence or at least decelerate disease progrediation. Moreover, the rise in absolute numbers of people inflicted far and wide proposes that management of main neurological disorders is globally inadequate. These people pose a significant socioeconomic burden owing to disability, illness and premature death [202, 203].

Unquestionably, inflammasome signaling is affected in several neurodegenerative disorders including AD, PD or HD that attract more increasing attention in studies investigating their place in clinical practice. Specifically, the NLRP3 inflammasome has been meticulously investigated in this context [204, 205], but recently, role of other inflammasomes as pathological drivers in brain diseases was also conceptualised by Anna Chiarini et al [46]. This attempt markedly indicates that there is an urgent need to extend our comprehension regarding neurodegenerative mechanisms associated with inflammasomes other than NLRP3.

**Figure 4.**
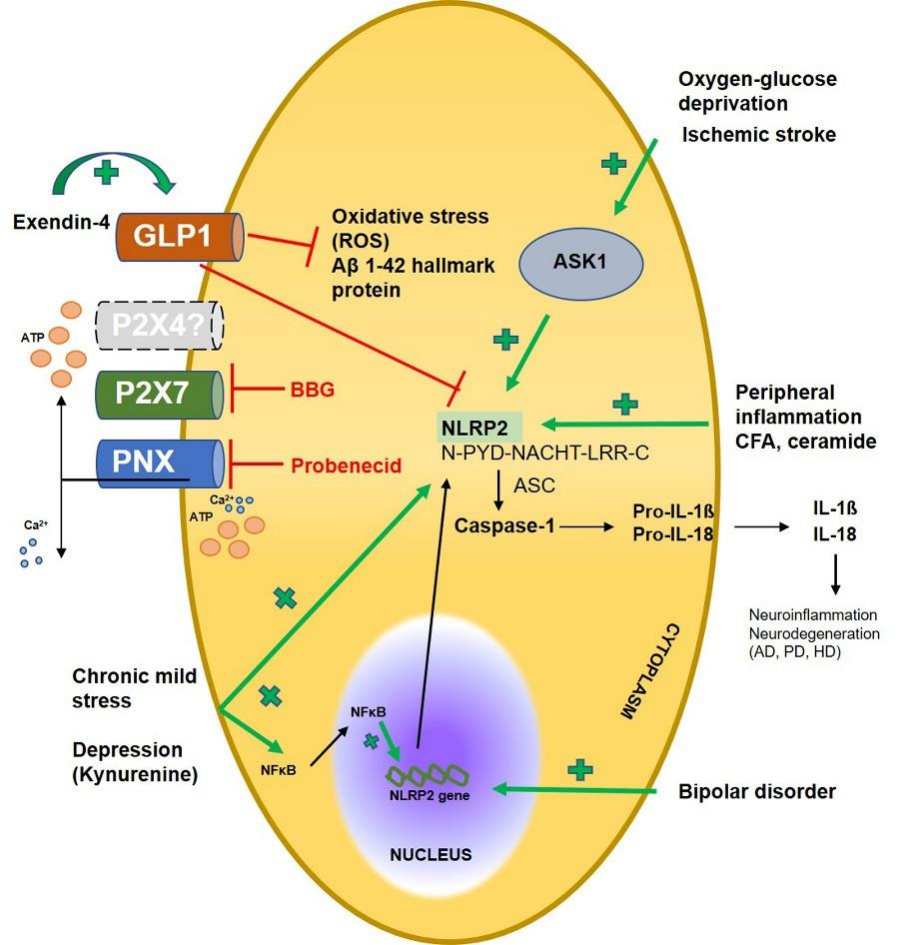
**State-of-the-art in relation to NLRP2 protein in neuropathologies**. ASC: Apoptosis-associated Speck-like protein containing a caspase-activation and recruitment domain, ASK1: Apoptosis signal-regulating kinase 1, BBG: Brilliant Blue G, GLP-1: Glucagon-like peptide-1, NFκB: Nuclear factor kappa B, P2X4, P2X7- P2X purinoreceptor X4,7, PNX1-Pannexin-1, ROS-reactive oxygen species.

First, in this study we attempted to encompass the literature focusing on the expression and distribution of NLRP2 inflammasome in human and rodent neuropathological disorders (Fig. 4). Taken together, in these models major NLRP2 expression was upregulated in primary astrocyte cultures [7, 32, 33, 34, 35], as well as in astrocytes of rodent spinal dorsal horn, respectively [31]. Beyond astrocytes, the role of NLRP2 was also verified in NeuN- and peripherin positive mechanical, but not thermal sensor subsets of DRG cells by Matsuoka et al [36]. Furthermore, tissue distribution of NLRP2 protein was observed not only in spinal cord, adult cortex, hippocampus or striatum [31, 32], but in neural stem cells as well [37].

Regarding activatory stimuli, particular attention is given to purinergic signaling in human NLRP2 activation, hence ATP is a well-known and adeptly characterised DAMP that facilitates inflammasome activation upon trauma [206]. Indeed, exogenous stimulation with ATP activates NLRP2 inflammasome resulting in caspase-1 mediated production of mature IL-1β [7]. According to all indications, ATP acts on P2X7 receptor that cooperates with PNX1, hence application of P2X7 receptor inhibitor BBG and PNX inhibitor probenecid diminishes NLRP2 activation. Nevertheless, it is important to underline that P2X4 receptor has been found to be functionally coupled with P2X7 receptor and pannexin-1 in NLRP3 inflammasome of gingival epithelial cells [207, 208]. Overall, these observations are in accordance with findings in which heme activates NLRP3 inflammasome in macrophages via P2X7 and P2X4 signaling during kidney inflammation [209]. We postulate a similar case with NLRP2 inflammasome, however there are no confirming experimental data. In our earlier article we hypothesized that overexpression of astroglial NLRP2 in spinal dorsal horn may be associated with significant P2X4 upregulation upon intraplantar CFA injection [31, 210]. Other triggering stimulus of NLRP2 activation is ischemic stroke, evoked by occlusion of middle cerebral artery or oxygen-glucose deprivation in astrocyte cultures [32]. Unsurprisingly, mounting evidence suggests that astrocyte-mediated inflammation may be potentially involved in the pathogenesis of mental disorders such as depression [211, 212]. In their recent study, Zhang et al [34] found that tryptophan metabolite Kyn, a specific biomarker of depressive behaviours, upregulated NLRP2 in astrocytes, supported by others also reporting significantly elevated levels of proinflammatory cytokines [213]. In bipolar disorder, neuroinflammatory biomarkers of cerebrospinal fluid were found to associate with cognitive decline. Moreover, cognitive impairment is increasingly recognised in people suffering from bipolar disorder, suggesting a link between neuroinflammation, neurodegenerative states and mood abnormalities [214], which may be significantly modulated by NLRP2 inflammasome as well [37, 45, 46].

Secondly, the present study collects the connectome of NLRP2, envisaged with STRING platform. The obtained set of proteins were further filtered to 15 possible protein interactors involved in brain disorders; their pathophysiological roles are discussed in details in chapter 4.1-4.15, TABLE S1. Many of these potential partnerships have not been earlier experimentally investigated. Besides signaling of NLRP2 inflammasome is also lacking from the collection of the Kyoto Encyclopedia of Genes and Genomes (KEGG) database Supplementary Figure 1.

Concluding the literature and STRING based matches we hypothesize that the filtered NLRP2 connectome influences a plethora of molecular processes in neurons, such as glutamatergic excitotoxicity, apoptosis/ survival signaling and neuroinflammation.

Glutamatergic excitotoxicity - The integrity of glutamatergic signaling is essential for preserving neuronal homeostasis and evading neurodegeneration. SLA1 prevents glutamatergic excitotoxicity by degrading excessive NMDA receptors [52]. As a synergist, apelin also disrupts NMDA receptor-mediated excitotoxicity in rat hippocampus through survival kinases AKT and Raf/ERK-1/2 [157]. Furthermore, the role of DLGAPs is to anchor glutamate receptors in the postsynaptic membrane and to link them with other proteins including other glutamate receptors, signaling- and cytoskeletal factors while regulating both ionotropic and metabotropic glutamate receptors via synaptic scaling [134-137]. Low *DLGAP2* expression was detected in age-related cognitive decline and AD [145], which may relate to other findings, such as low spine density or reduced excitatory postsynaptic current, revealed in orbitofrontal cortex of *DLGAP2* knockout mice [140, 142]. Directed expression bHLH transcription factor NEUROD1 stimulates neuronal maturation as well as ameliorates deficits of dendritic spine density in hippocampal neurons of APPxPS1 model of AD [111]. Besides, ENT1 inhibitor J4 mitigates the damages of long-term potentiation, excitatory synaptic expression as well as GSK-3ß or PKA signaling of neuronal plasticity and alleviates memory deficits in APP/PS1 model of AD [176].

Apoptosis/survival signaling- It is of particular importance to gain a better understanding of the signaling of neuronal apoptosis/survival in neurodegenerative states. Impairment of autophagy via reduction of BECN1 level results in overproduction of microglial IL-1β and IL-18 proinflammatory cytokines in AD patients. Deficits of BECN1 mediated phagocytosis causes dysfunctional recruitment of phagocytic receptors that may associate with extracellular accumulation of Aβ plaques and other cellular debris. Recently, in early phase of HD BECN1 administration has successfully cleared mutant HTT accumulation and reversed progrediation [59-62]. Apelin exerts positive impacts on redox homeostasis and prevents mitochondrial cytochrome c release and caspase-3 activation in cultured murine cortical neurons [157].

Furthermore, EPS15 participates in the EGF-AKT pathway mediated pro-survival cell signaling reduced in dopaminergic neurodegeneration [98, 99]. CCDC50 autophagy receptor inhibits inflammatory responses by disrupting NLRP3 [197, 198] and prevents EGFR downregulation as well as regulate NF-κB, Fas and interferon signaling. As exposure, in contrast, triggers apoptosis of cerebellar neurons via activation of JNK and p38MAPK signaling [182] as well as upregulates Bax and decreases Bcl-2 factor [192]. A rescue factor SUGT1, functioning as a chaperone protein protein, was also recognised to counteract pathological aggregation of α-synuclein and neurotoxicity in PD. *SUGT1* mRNA was highly elevated in fronto-temporal cortex in human PD [90, 91]. Its significance has also been earlier described in AD, hence decreased SUGT1 immunopositivity was found in degenerating neurons of AD patients [92]

Dysregulation of aforementioned GSK-3 signaling, inhibited by PDK1/AKT kinase [215, 216], contributes to the hyperphosphorylation of tau protein as well as Aβ-induced cell death in AD pathogenesis. Abundant GSK-3 level was found in postmortem brain of AD patients, moreover large body of evidence support that GSK-3 activator lysophosphatidic acid associates with AD biomarkers Aß, total tau and phospho-tau [72, 74]. In contrast, GSK-3 inhibits ER stress sensor γ-TXLN, promoting apoptosis- and autophagy. In addition, blockade of γ-TXLN alone may lead to tau hyperphosphorylation in AD [131].

Neuroinflammation- Several key mechanims have been identified in neurodegenerative diseases as summarized above, also including neuroinflammation, which evokes a great challenge to clinical practice. Neuroinflammation has been recognised in dementia and is typically linked to cognitive decline with elevated levels of proinflammatory markers (IL-1, IL-6, IL-8, C-reactive protein) in patients suffering from dementia [217-219]. However, recent data suggest that inflammatory proteins may express both pro- and anti-inflammatory actions making the interpretation even more difficult in complex neurodegenerative states [220]. In fact, the versatility of postmortem samples as well as challenges of appropiate resolution in detection of cytokines may all lead to controversial conclusions [221, 222]. Indeed, neuroinflammation is still regarded as one of the crucial molecular processes of dementia, but we still do not know whether this mechanism is consequential or causative, in regards of neurodegenerative progrediation. Of note, it receives growing evidence that neuroinflammation may appear as an early temporal red flag event that may link to other mechanisms contributing to neuropathologies [223-225].

In fact, neuroinflammation and generally the therapeutic potential of targeting inflammasomes has been increasingly recognised in neurodegenerative conditions since 2013, when Heneka et al. [226] proved the significance of NLRP3 inflammasome with *NLRP3* -/- mice in AD. Since then, many efforts have been made to seek selective and potent NLRP3 inhibitors, because the currently US Food and Drug Administration (FDA) approved inhibitors of multiple inflammatory diseases include only canakinumab, anakinra and rilonacept. Nevertheless, these inhibitors do not cross efficiently the blood-brain barrier and lack proper pharmacokinetic properties [227, 228].

A great achievement was reached when a potent diarylsulfonylurea compound MCC950 (also termed as CRID3, CP-456773), endowed with NLRP3 selectivity, showed therapeutic improvement in several preclinical models such as experimental autoimmune encephalomyelitis, such as AD and PD [229-232], nevertheless it failed in clinical trials because of off-target toxicity.

In the search for new inhibitors Stavudine (d4T), acting as an inhibitor of nucleoside reverse transcriptase, has been published recently to downregulate NLRP3 activation in AD [233]. Furthermore, Gastaldi et al. [234] by applying the pharmacophore-hybridization method synthesised several benzo[d]imidazole-2-one derivatives to test their inhibitory effect on NLRP3 evoked pyroptosis and IL-1β production.

Epigenetic research has also recently provided ammunition against inflammasomes. Three molecular events have come into focus: CpG DNA methylation, posttranslational modification of histones as well as noncoding RNA expression. From this point forward microRNAs may be promising therapeutic targets. Currently, as NLRP3 represents the most investigated inflammasome till date, testing of miR-30e and miR-7 have already started against this inflammasome type in PD [235, 236, 237].

However, it is increasingly becoming clear that NLRP3 is not the only inflammasome involved in neurodegenerative states. Notably, Kaushal et al. [238] has already reported elevated mRNA level of *NLRP1* and highlighted the causative role of NLRP1-caspase 1-caspase-6 signaling in the accumulation of Aβ_42_ deposits in AD.

For clarity, we need to examine those common reasons why proper therapeutic options targeting human inflammasomes including NLRP2 are still limited till date:

- There are theories and evidence that inflammasomes can functionally take over each other’s roles, albeit these mostly unrevealed interplays among canonical and/or non-canonical inflammasomes need to be further investigated. Denes et al. [29] showed in their rodent model that NLRC4 (NLR family, CARD domain containing 4) and AIM2 contribute a lot to the pathogenesis of acute ischemic brain injury in the presence of pharmacologically blocked NLRP3.
- Unfortunately, it has been confirmed that the genetic signature as well as organs of rodents and humans differ greatly. This inter-species discrepancy also applies to the architecture of CNS, as in the most used rodent models usually only the structurally less complex olfactory cortex was put under scrutiny. Furthermore, even at a cellular level many morphofunctional aspects differ between humans and rodents [46, 239], and if that is not enough of a problem specifically their inflammasome regulation is also markedly distinct [240, 241]. Thus, even though we have a lot of animal experiments available from animals’ models, low efficiency rate of rodent-human translation has been delaying the development of breakthrough drugs for brain disorders for decades.
  Currently, there is only one promising “game changer” in this struggle we can carry, the Lecanemab, IgG1 monoclonal antibody, which has been recently proven to reduce amyloidosis and cognitive deterioration in early-stage AD patients compared with placebo group, but we cannot forget about its unpleasant side effects such as brain swelling or hemorrhage [242].
- Above that, even myriad of inhibitors is mentioned with potential therapeutic benefits against inflammasomes like NLRP3 [46, 227, 228, 229], these inhibitions mostly result from blocking related signaling pathways (such as dopamine receptor [243], adiponectin receptor [244], estrogen receptor [245], Angiotensin II receptor [246]) rather than direct inhibitory strategies. Thus, the same may be the case with NLRP2, if the major signaling pathways are precisely revealed. Data mining techniques like STRING can help us to target directly the potentially NLRP2-associated protein candidates and test them experimentally.
  Nevertheless, it has been suggested to reduce the function of not only NLRP3 but all canonical inflammasomes by inhibiting the ASC protein with a newly developed small molecule inhibitor MM01 [247].
- Finally, despite accumulating experimental evidence, unfortunately, few experts in the field still take seriously the idea that the rodent and human NLRP2 inflammasome should be addressed more seriously.

## 6. Conclusions

Taken together, based on STRING analysis human NLRP2 inflammasome may cooperate with proteins that are related to excitoxicity, cellular apoptosis/survival and neuroinflammation. Thus, this filtered connectome in the nervous system requires an experimental approach both in rodent and human models. In the future, complete understanding of inflammasome signaling including NLRP2 mediated processes may open up new prospects for efficient inhibitors and/or therapeutic agents, which are currently lacking from our armamentarium.

## Supplementary Materials

The Supplementary data can be found online at: www.aginganddisease.org/EN/10.14336/AD.2021.0926-1.

Author Contributions Conceptualization: LD Manuscript writing, data visualisation, editing and supervision: LD, BG. All authors have read and agreed to the published version of the manuscript.



## Data Availability

The data sets and materials supporting the conclusions of this study are included within the article.
