# Supplementary material for: The Neglected Sibling: NLRP2 Inflammasome in the Nervous System
Source: Aging Dis. 2024 May 7;15(3):1006–28. doi: 10.14336/AD.2023.0926-1 (PMC11081174; doi:10.14336/AD.2023.0926-1)
Supplement: Supplementary file 1 [file AD-15-3-1006-s.pdf]

SUPPLEMENTARY DATA

The Neglected Sibling: NLRP2 Inflammasome in the Nervous System

László Ducza[#*], Botond Gaál[#]

© 2023. Ducza L et al. Published online at http://www.aginganddisease.org/EN/10.14336/AD.2023.0926-1

SUPPLEMENTARY DATA

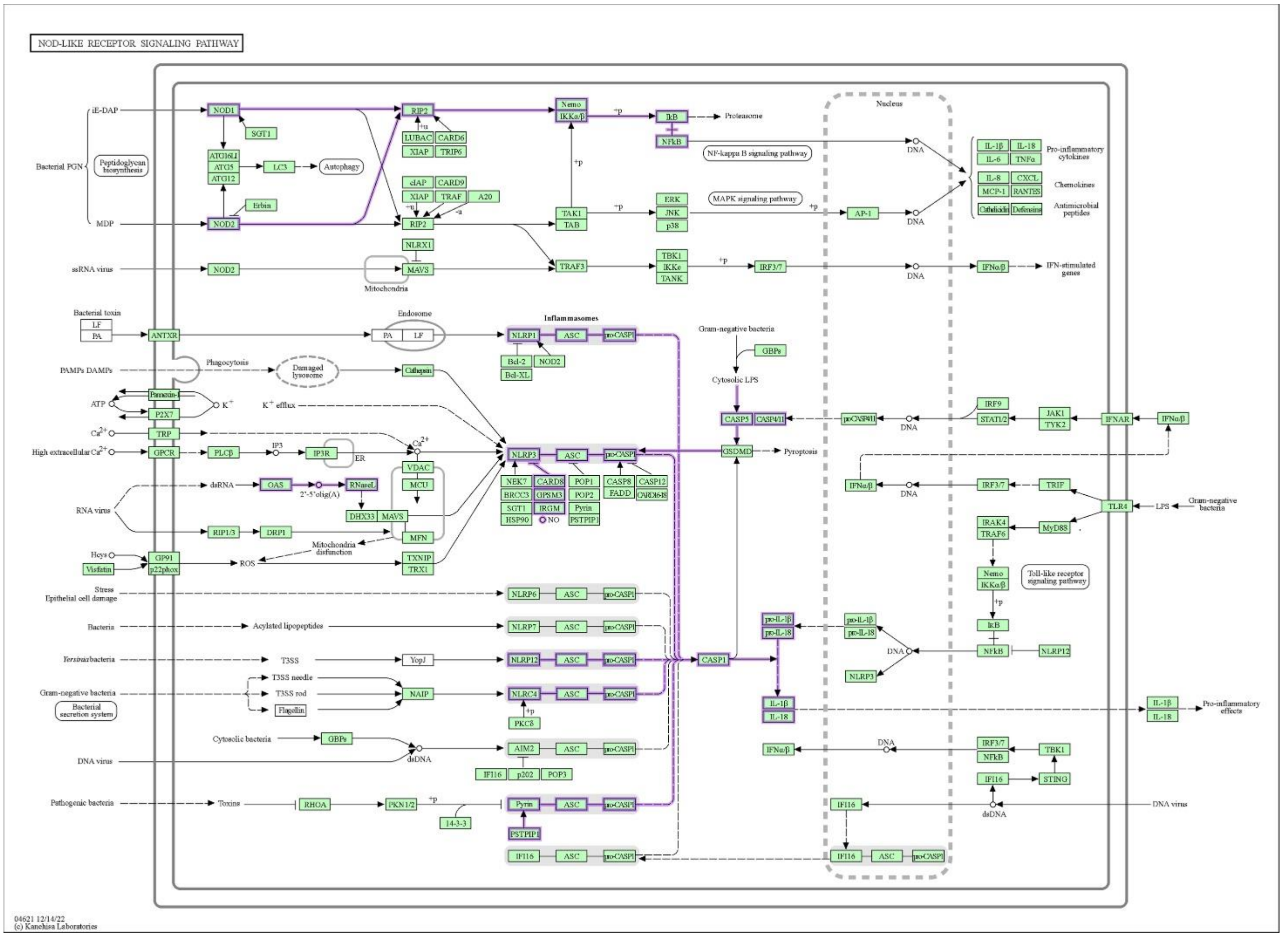

Supplementary Figure 1. Available Nod-like receptor signaling pathways in KEGG database.

Supplementary Table 1. List of NLRP2 interactors involved in neuropathologies (in order of interaction score)

	Node protein hits	Full name	Interaction score
**1**	SLA	Src-like-adapter;Adapter protein	0.752
**2**	BECN1	Beclin-1	0.664
**3**	PCDH11X	Protocadherin-11 X-linked; Potential calcium-dependent cell-adhesion protein	0.547
**4**	GYG2	Glycogenin-2	0.515
**5**	MRE11A	Double-strand break repair protein MRE11	0.490
**6**	SUGT1	SGT1 homolog, MIS12 kinetochore complex assembly cochaperone	0.485
**7**	EPS15	Epidermal growth factor receptor substrate 15	0.468
**8**	NEUROD1	Neurogenic differentiation factor 1	0.466
**9**	CAPN6	Calpain-6	0.462
**10**	TXLNG	Gamma-taxilin	0.455
**11**	DLGAP2	Disks large-associated protein 2	0.449
**12**	APLNR	Apelin receptor	0.445
**13**	SLC29A1	Solute carrier family 2	0.444
**14**	AS3MT	Arsenite methyltransferase	0.423
**15**	CCDC50	Coiled-coil domain-containing protein 50	0.421

© 2023. Ducza L et al. Published online at http://www.aginganddisease.org/EN/10.14336/AD.2023.0926-1
